# The importance of historically popular sires on the accuracy of genomic predictions of young animals in the US Holstein population

**DOI:** 10.3168/jdsc.2022-0299

**Published:** 2023-04-20

**Authors:** Yvette Steyn, Thomas J. Lawlor, Daniela Lourenco, Ignacy Misztal

**Affiliations:** 1 Department of Animal and Dairy Science, University of Georgia, Athens 30602; 2 Holstein Association USA Inc., Brattleboro, VT 05302

## Abstract

The dairy industry is known for its extensive use of artificial insemination, which has resulted in a population where most animals can be traced back to only a few sires. Due to their relatedness to the population, old influential sires could still contribute to the accuracy of genomic predictions. The objective of the study was to identify the impact of historically influential sires on the recent population. This was tested by constructing a genomic relationship matrix using recursion with different sets of sires. Differences in prediction accuracies with different sets are indicative of how important each set is. Recursion coefficients linking young animals to those sets reveal the relative importance of specific sires to the prediction accuracy of recent animals. The data included ∼10 million scores for stature and fore udder attachment (FUA) measured from 1983. Genotypes of 569,404 animals were available. Sire sets included the 100 most popular sires born within different time periods. Computations were with single-step genomic BLUP. In general, the younger sires had higher prediction accuracies than the oldest sires, even though they generally have fewer progeny. The accuracy of evaluation for stature was increased from 0.54 with the most popular sires born before 1981 to 0.69 with sires born from 2001 to 2010, while the accuracy for FUA increased from 0.47 to 0.61. The accuracy achieved using the overall 100 most used sires was 0.66 for stature and 0.58 for FUA. All 100 sires from each period were combined in a subset to determine the importance of each sire relative to all 400 animals in the combined subset. The highest relative impact of a sire that was born within the different time sets was 1.97 for Valiant (before 1981), 1.94 for Blackstar (1981 to 1990), 4.38 for Shottle (1991 to 2000), and 3.09 for Planet (2001 to 2010). The 3 sires among the 400 with the greatest impact were Shottle, Goldwyn (3.73), and Planet. The relative impact of a sire was not strongly related to the number of progeny. For instance, the relative impact of Durham with 34K progeny was 2.29, whereas the impact of O Man with 15K progeny was 3.13. The impact of a sire is also influenced by whether it was used as a sire of sires. Results show that younger sires are more relevant to the accuracy of breeding value prediction in the recent population.

## Highlights

• Historically popular sires capture the genetic composition of recent populations, but younger sires capture more.
• Sires with the greatest number of progeny did not necessarily have the greatest genetic contribution to the current population.
• Genotypes of old sires can be removed from evaluations.

## Graphical Abstract

**Figure:**
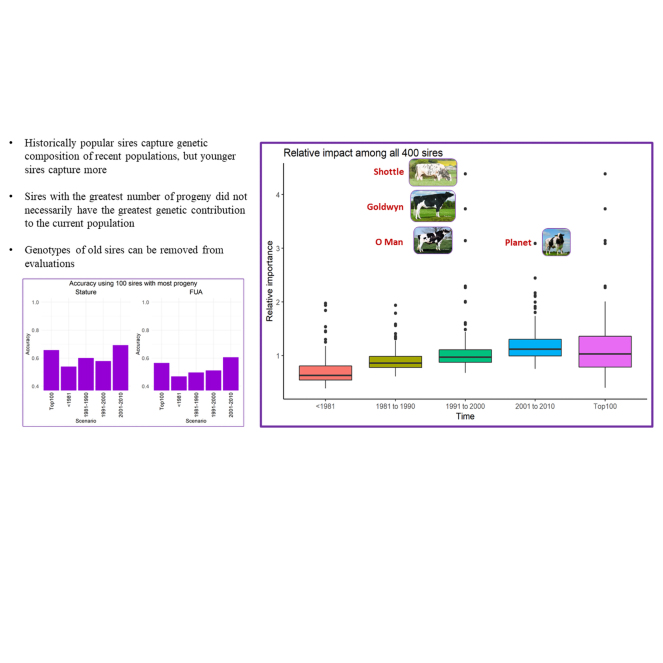
**Summary:** The US dairy industry is known for its extensive use of reproductive biotechnology, which has allowed a small number of bulls to have a disproportionately large genetic contribution to the population. The objectives of this study were to investigate whether historically extensively used sires still contribute to the accuracy of genomic predictions of young animals. Results show that old sires still capture some of the genetic variation of recent populations, but contribute less to the accuracy of prediction compared with more recently used sires. The sires that were identified as genetically more important (Shottle, Goldwyn, and Planet) did not have the highest number of registered progeny.

The dairy industry is known for its wide use of a relatively small number of bulls. Yue et al. (2015) found that all AI bulls in the United States in 2015 were descendant from only 2 bulls born in 1880. These lineages were mostly distributed in the population through 3 extensively used bulls, namely Pawnlee Farm Arlinda Chief (Chief), Round Oak Rag Apple Elevation (Elevation), and Penstate Ivanhoe Star (Ivanhoe Star) (Yue et al., 2015). The results of their study were based on tracing the Y-chromosome, which is a small fraction of the genome. However, evaluating autosomal chromosomes would include variation from both male and female sources. For many years, the Holstein sire summaries have reported that Elevation and Chief have had the highest proportion of genes in common with the available proven bulls when based on pedigree relationships. Historically popular sires were the best animals at the time. However, these previously superior sires are expected to compare unfavorably with modern animals based on net merit and production traits (De Vries, 2017), since remarkable genetic progress has been made over generations of selection. A recent study aimed to re-incorporate lost genetics from older bulls to improve both diversity and fitness. Progeny resulting from these old bulls showed average or better performance for daughter pregnancy rate, but below average for production traits (Dechow et al., 2020). This is evidence that many decades of strong selection created a modern animal that is considerably different compared with historic populations, both phenotypically and genotypically. Considering this difference, the relative importance of old bulls could be lower than modern animals. Although the above-mentioned sires may be related to almost all modern animals, they are not necessarily strongly related. Parent contributions halve in every generation, which makes a specific parent irrelevant to descendants decades later. However, in the dairy industry, popular sires breed sons that also become popular sires. Additionally, they are used over long periods of time instead of simply one generation. This may increase their genetic representation in later generations.

The breeding values of animals can be conditioned on the breeding values of a subset of animals based on the relationships between this subset and all remaining animals using a recursive formula (Misztal et al., 2014a). When the subset size is large enough (∼15K in US Holstein), the prediction accuracy is similar to using all animals, regardless of the choice of animals (Fragomeni et al., 2015). When the subset is small, differences in accuracy are indicative of the relevance of the subset. The recursion coefficients indicate the impact of specific bulls. The objective of this study was to determine whether old, influential sires have greater genetic contributions to the prediction of genomic breeding values of young animals compared with popular sires with fewer progeny or sires born more recently.

Institutional animal care and use or equivalent approval was not required since data were already recorded by the industry. The data were obtained from the Council on Dairy Cattle Breeding (**CDCB**) and contained information up to 2014. It included 9,817,252 animals in the pedigree, of which 330,837 were sires and 5,471,039 were dams. Phenotypes started in 1983 and included 6,550,442 animals with 10,067,745 measures for stature and 10,067,730 for fore udder attachment (**FUA**). These traits were used because they have shown to be type traits with substantial genetic change over time (Tsuruta et al., 2021). There were 569,404 genotypes with 58,990 SNP markers. This included 121,634 genotyped males, of which 21,824 were sires and 15,908 were sires with phenotyped progeny. The genotyped sires included 8,730 animals with more than 50 progeny, with the maximum number of progeny being 58,266 from Marshfield Elevation Tony (Mars).

The traditional BLUP evaluation depends on the recursion equation

$$u_{i}=\frac{\left(u_{sire}+\;u_{dam}\right)}{2}+m_{i},$$

where *u_i_* is the breeding value of animal *i*, *u_sire_* and *u_dam_* are breeding values of the parents, and *m_i_* is the Mendelian sampling of animal *i*. The impact of ancestors is halved in every generation, making old sires less important. Genomic evaluations, whether by genomic BLUP (GBLUP) or indirectly with SNP-BLUP, depends on an extension of this equation to reflect the genomic relationships between animals. The extended equation to condition the genomic breeding values of animal *i* on the genomic breeding values of the subset animals *j* is $u_{i}=\underset{j\;\in n}{\sum }\;p_{ij}u_{j}+\;\phi_{i}$ for *i* > *n*, where *p_ij_* is a relationship between animal *i* and subset animal *j*, *u_j_* is the breeding value of the animal *j*, $\phi_{i}$ is uncaptured Mendelian sampling in the breeding value of animal *i*, and *n* is the size of the subset (Misztal et al., 2014a). In the recursive equation, individual animals in the subset may have different impacts on the accuracy of prediction of the remaining animals. A large *p* coefficient indicates greater impact.

In this study, the genomic relationship matrix (**G**) was obtained using all genotyped animals and the formula

$$G=\frac{MM^{\prime }}{2\;\sum p_{i\;}\left(1-p_{i\;}\right)},$$

where **M** is a matrix of SNP content centered by twice the current allele frequencies, and *p_i_* is the allele frequency for SNP *i* (VanRaden, 2008). To reduce bias due to the different genetic level of genotyped and nongenotyped animals, **G** was tuned to be compatible with the pedigree relationship matrix between genotyped and nongenotyped animals (**A22**) using the method described by Chen et al. (2011). To avoid singularity, 5% of A22 was combined with 95% of **G**.

The inverse of the resulting genomic relationship matrix can be approximated by using a subset as the core animals in the algorithm for proven and young (APY) (Misztal et al., 2014a). Five different subsets (cores) of 100 sires were used based on their birth year and the total number of registered progeny as of 2014. These different subsets were the most used sires overall (top 100) regardless of when the sire was born, and the most used sires born within different time periods: before 1981, 1981 to 1990, 1991 to 2000, and 2001 to 2010. To allow comparison of the impact of popular sires over all time periods, all 400 were combined in a subset. The relative impact of an animal was calculated as

$$\frac{n\sum_{j=1}^{m}\;\left|p_{ij}\right|\;}{\sum_{i=1}^{n}\;\sum_{j=1}^{m}\;\left|p_{ij}\right|\;},$$

where *n* is the number of animals in the subset, *i* is the individual sire, *m* is the number of validation animals, *j* is the validation animal, and *p_ij_* is the recursion coefficient corresponding to the 400 animals in the combined subset and the validation population.

Stature and FUA were analyzed in a 2-trait model as described by Tsuruta et al. (2021). Single-step GBLUP (**ssGBLUP**) was used to accommodate genotyped and nongenotyped animals with the BLUPf90iod2 package within the blupf90 software suite (Misztal et al. 2014b). The pedigree depth was 30 generations, starting from the phenotyped animals. A traditional pedigree-based BLUP was used to obtain adjusted phenotypes (y_adj_) using the PREDICTF90 software package (Misztal et al., 2014b). These are phenotypes adjusted for all effects in the model other than the animal effect. Validation animals were genotyped animals born after 2010 with only one measurement, which totaled 10,153 animals. The empirical prediction accuracy was measured as the Pearson correlation between the genomic breeding value and y_adj_ divided by the square root of the heritability (0.45 for stature and 0.18 for FUA).

Figure 1 shows the accuracies obtained when only the 100 most used sires in different time periods were used as a subset. Although using the overall 100 most popular sires also delivers high accuracies, it is still lower than using only sires born from 2001 to 2010. The accuracies obtained when using sires born before 1981 are the lowest (0.54 for stature and 0.47 for FUA). This is still an impressive accuracy reached when only 100 animals are used for the conditioning of the inverse of the genomic relationship matrix of the remaining population. This indicates that these old sires still capture independent chromosome segments (**ICS**) of the current population. The highest accuracies were reached using the 100 sires from the period 2001 to 2010 (0.69 for stature and 0.61 for FUA). This is approximately 30% more than using the older sires. Thus, the ICS are better captured by young sires. It has been shown that the accuracy of genomic breeding values is influenced by the generational distance between the reference and validation population (Hidalgo et al., 2021; Hollifield et al., 2021). Although the old sires may be related to the majority of the current population, they are not highly related. Many generations of recombination have occurred between old sires and the recent population. Consequently, they may share smaller haplotypes with the validation population and different epistatic effects. Allele substitution effects can differ between the modern population and ancestral population due to other nonadditive effects and different allele frequencies (Legarra et al., 2021). In contrast, young animals are closely related to the validation population and share larger haplotypes.

**Figure 1:**
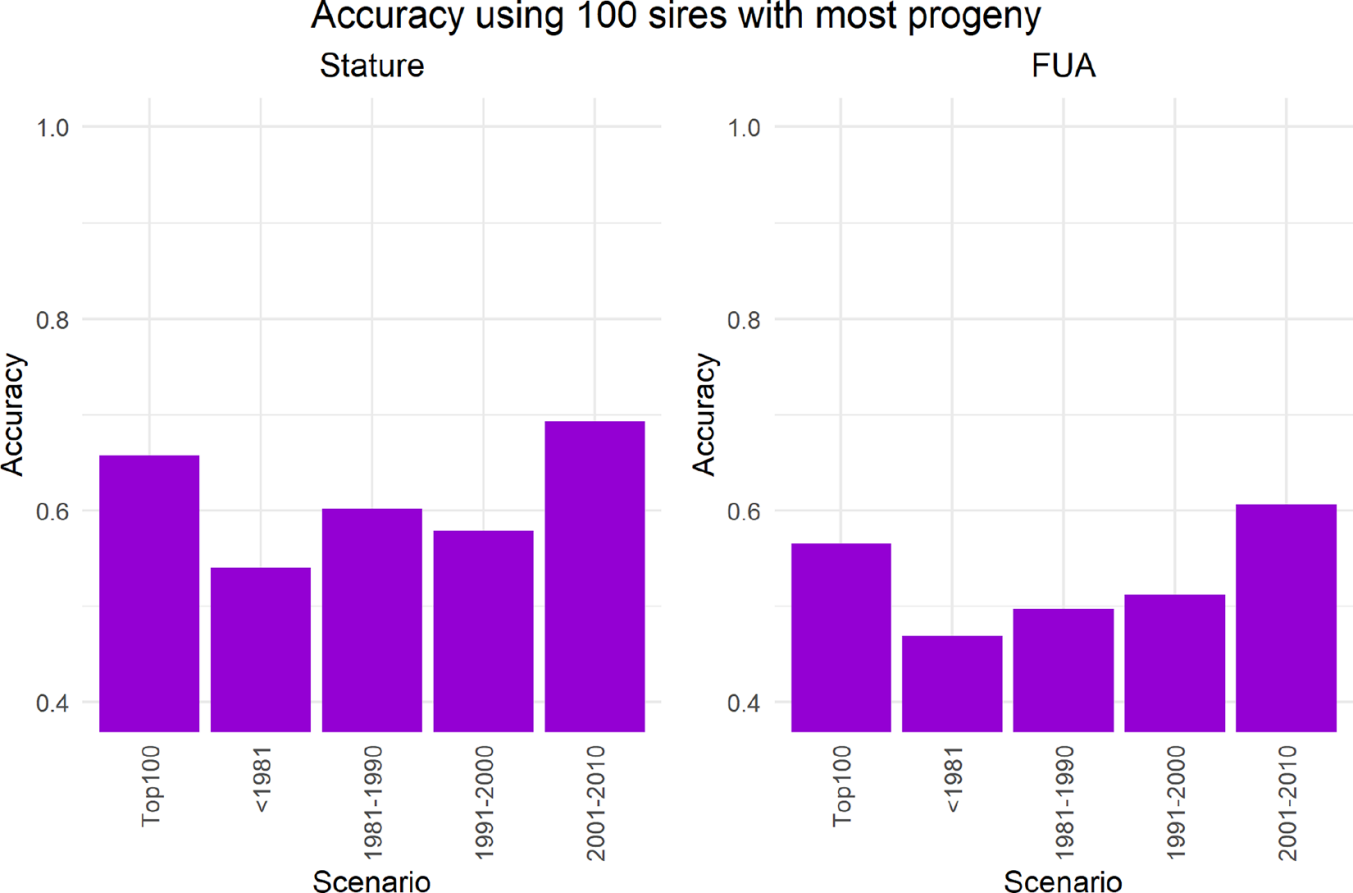
The accuracy of prediction for stature and fore udder attachment (FUA) when using a core size of 100 sires. The different cores included the 100 sires with the most progeny in the populations, regardless of birth year (Top100), and the 100 most used sires born within different 10-yr time periods.

Figure 2 shows boxplots of the relative impact of sires within each 100-animal subset when the recursion coefficients are based on all 400 animals combined, and the validation population. This makes all 400 animals comparable across groups. Table 1 provides the names and information of the 4 most important sires within each time period and overall. The 3 highest outliers are within the time period 1991 to 2000, with the top 3 being Picston Shottle (Shottle), Braedale Goldwyn (Goldwyn), and O-bee Manfred Justice (O Man). The total number of progeny of an animal contributes to its genetic impact on the population, but is not the best indicator of true genetic impact. Many highly popular sires were used in both registered and commercial herds as a sire of dams, while specific sires were used more frequently as a sire of sires. Mars is the bull with the most progeny in the overall population, yet he does not appear among the top 4 most important sires in his time period. Madawaska Aerostar (Aerostar) is the second most important sire within the time period 1981 to 1990, even though he only had 9,805 progeny—considerably fewer than the 42,291 progeny of the most important in that time period (To-mar Blackstar). However, Aerostar is the maternal grandsire of the 2 most important sires overall: Shottle and Goldwyn.

**Figure 2:**
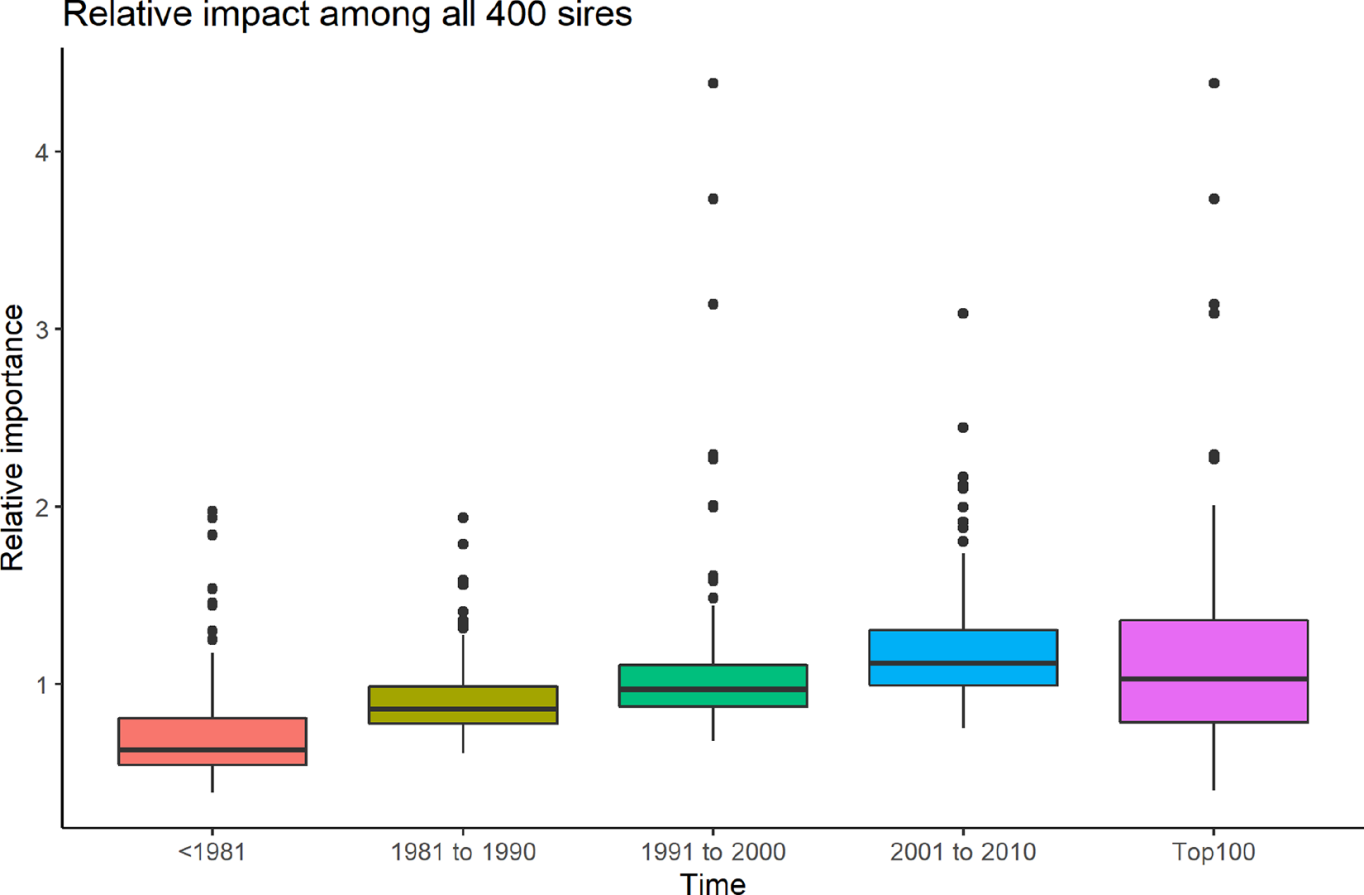
The relative importance of the 100 sires within each time period on the accuracy of genomic prediction of the validation population. The line above the box is the first quartile of the data (up to 25th percentile), the upper part of the box is the second quartile (25th to 50th percentile), the line in the box is the median, the lower part of the box is the third quartile (51 to 75 percentile), and the line below the box is the fourth quartile (76 to 100th percentile). The dots indicate outliers that are outside 1.5 times the interquartile range from the 25th to 75th percentile, starting from the edge of the box.

**Table 1:** The 4 sires born within specific time periods with the greatest relative impact on the recent population, along with their sire and maternal grandsire (MGS)^1^

Time	Name	Sire	MGS	Birth year	Impact	Progeny	Registration ID
Before 1981	SWD (Valiant)	Chief	Admiral	1973	1.97	37,939	HOUSAM1650414
	Carlin-M Ivanhoe (Bell)	Ivanhoe Star	Heilo Bell	1974	1.94	51,364	HOUSAM1667366
	Walkway Chief (Mark)	Chief	Fond Matt	1978	1.84	44,553	HOUSAM1773417
	Round Oak Apple (Elevation)	B. Elevation	Ivanhoe SH	1965	1.54	39,438	HOUSAM1491007
1981 to 1990	To-mar (Blackstar)	Chairman	Wayne	1983	1.94	42,291	HOUSAM1929410
	Madawaska (Aerostar)	Starbuck	Majesty	1985	1.79	9,805	HOCANM383622
	Emprise Bell (Elton)	Bell	Arlinda	1983	1.59	11,395	HOUSAM1912270
	MJR Blackstar (Emory)	Blackstar	Mark	1989	1.56	18,443	HOUSAM2114601
1991 to 2000	Picston (Shottle)*	Mtoto	Aerostar	1999	4.38	14,935	HOGBRM598172
	Braedale (Goldwyn)*	James	Aerostar	2000	3.73	11,291	HOCANM10705608
	O-Bee Manfred Justice (O Man)*	Manfred	Elton	1998	3.13	15,515	HOUSAM122358313
	Regancrest Elton (Durham)	Elton	Mark	1994	2.29	34,267	HOUSAM2250783
2001 to 2010	Ensenada Taboo (Planet)*	Taboo	Amel	2003	3.09	14,038	HOUSAM60597003
	Lady's Manor PL (Shamrock)	Planet	Shottle	2009	2.17	7,880	HOUSAM68977120
	Desu (Observer)	Planet	O Man	2008	2.12	4,670	HOUSAM65917481
	Regancrest (Altaiota)	O Man	Ito	2005	2.11	5,248	HOUSAM61898306

1 These sires were among the 100 most used sires born within different time periods. The relative impact is centered around the average when all 400 animals are combined in the subset on which recursion is based. The bull's commonly used short name is shown in parentheses.

* The 4 individuals with the greatest importance compared to all 400 bulls, regardless of when they were born.

Elevation, Chief, and Ivanhoe Star are responsible for the limited number of Y-chromosome lineages in North American AI sires (Yue et al., 2015), yet only Elevation is among the top 4 most important sires before 1981. Elevation is 1.54 times more important than the average of the 400 selected sires, which is far lower than Shottle, which is 4.38 times more important. Although Chief and Ivanhoe Star do not appear among the top 4, Chief is the sire of 2 of the top 4 (Mark and Valiant), while Mark is the maternal grandsire of Durham—the fourth most important sire born within the period 1991 to 2000. Ivanhoe Star is the sire of Bell (the second most important before 1981). He has sired Elton, which is the sire of Durham and the maternal grandsire of O Man. O Man was commonly used to improve health and fitness qualities. Goldwyn is known as a bull with outstanding conformation qualities, while Planet has been popular for his production merit. The Holstein Association USA regularly releases a sire summary, which also lists the bulls with the highest average percentage of genes in common with the available proven bulls. In December 2014, Shottle ranked third, O man twelfth, Goldwyn fifteenth, and Planet seventeenth. By August 2022 when the young sires had the opportunity to produce more proven bulls, O man ranked third, Planet fifth, and Shottle sixth, while Goldwyn was outside the top 20. The top 2 bulls were Elevation and Chief.

Since younger, popular sires capture more of the genetic variability in the recent population based on our study, it may not be necessary to still include historical records in genetic evaluations when many genotypes are available. It has been shown that truncating pedigree information beyond 3 generations does not decrease the accuracy of prediction for recent animals (Lourenco et al., 2014). Generational distance between training and target populations has also shown to lead to a decay in accuracy of genomic predictions in poultry and pig populations (Hidalgo et al., 2021; Hollifield et al., 2021). Clear visual differences can be observed when comparing modern animals of a breed to those alive decades ago when strong selection has been applied. A comparison of productivity of the Holstein population in 2007 and 2017 showed that the same unit of energy-corrected milk could be produced using 74.8% of the cattle, 82.7% of the feedstuffs, 79.2% of the land, and 69.5% of the water (Capper and Cady, 2020). When dairy animals in 2007 were compared with dairy in 1944, 21% of animals, 23% of feedstuffs, 35% of the water, and only 10% of the land were required to produce 1 billion kilograms of milk (Capper et al., 2009). This remarkable progress is not due to management and nutrition alone. Genetic trends in the US Holstein have shown that production traits have improved favorably while fitness traits have deteriorated (De Vries, 2017). Changes in allele frequencies of the Holstein population in the 1960s have also been observed compared with the population in the 2010s and attributed to genetic selection (Ma et al., 2019). Considering these differences, information of old animals is irrelevant. In practice, most available information is included in genetic evaluations (with or without genomic information), whether they are relevant for prediction of current selection candidates or not. The main purpose is to present genetic trends to quantify and visualize genetic change over time. A compromise between disregarding old information and using all available information would be to remove only their genotypes or reduce the time period for genetic trends (or both).

The dairy industry has a unique breeding structure where single bulls have many offspring that, in turn, may have many offspring. The sire with the most progeny in this data set (Mars), had fewer than 60,000 direct progeny. However, popular dairy sires are known to have hundreds of thousands of progeny. The bulk of the sires' offspring are in herds that do not participate in US animal improvement programs, such as US commercial herds and foreign herds.

The “Millionaires Club” refers to sires that have produced over 1 million semen doses. One member is Jenny-Lou Mrshl Toystory (Toystory), born in 2001. At the time of his death in 2015, he had produced more than 2 million semen doses that were used in more than 20 countries. Despite his commercial popularity, he only had 14,726 registered progeny in the United States as of 2014. Based on our results, Toystory's relative impact ranks at 90 among the 100 sires with the most registered progeny. Currently, Toystory has more than 162K total daughters with milk production records in over 27 countries, of which ∼45K are recorded in the United States (CDCB, 2022). Due to the lack of information from these commercial and foreign herds, the true impact of specific sires toward the gene pool of the whole US population cannot be determined.

This study was limited to conformation traits, which are generally less commonly measured than production. As of 2022, O Man had a total of 61K daughters measured for production in the United States but only 15K for type traits (CDCB, 2022). Therefore, the effect of sires on the accuracy of prediction may be different for other traits. However, the relative importance of sires to capture the ICS of the population will be the same for all traits since genetic relationships are trait-independent. Additionally, this study used only information from registered US animals, while bulls often have many (if not most) daughters measured abroad. For example, as of 2022, O Man had a total of 103K daughters measured for production worldwide, of which 61K were in the United States. Goldwyn had 9K US daughters measured for production in the United States, but a total of 90K worldwide. Shottle had 19K in the United States but 147K worldwide (CDCB, 2022). Therefore, the impact of these bulls go beyond the US border and their relative importance may differ in other countries.

In conclusion, historically popular bulls still capture some of the ICS in the recent population, but have a relatively low impact on the accuracy of predictions compared with younger sires. Consequently, including their information in genetic evaluations may not be necessary. The total number of progeny a sire has is not the only determinant of importance in the population. Specific bulls can have a greater impact when they are used as a sire of sires. The true impact of sires on the US Holstein population cannot be determined due to the lack of participation of commercial herds.
